# Variation in Bird Gut Microbiota Across Dietary Guilds and Migratory Strategies

**DOI:** 10.1002/ece3.73879

**Published:** 2026-09-07

**Authors:** Wenjing Zhou, Yuan Lin, Ziwen Liu, Yajing Ma, Shufeng Hu, Junwu Huang, Zhou Lu, Lijiang Yu

**Affiliations:** ^1^ Animal Science and Technology College Guangxi University Nanning China; ^2^ Nanning Wuxu International Airport Co., Ltd Nanning China; ^3^ Guangxi Zhuang Autonomous Region Forestry Research Institute Nanning China

**Keywords:** birds, feeding habit, microbiome, migratory strategies

## Abstract

Birds have a unique physiology, and their gut microbiota is regulated by ecological factors such as diet and migratory strategies. However, our understanding of how these factors drive the assembly and changes in the gut microbiota in birds is limited. We performed 16S rRNA sequencing of 61 bird cloacae samples. The results showed significant differences in the composition of the gut microbiota among birds with different ecological types (*p* < 0.05), and each taxonomic group (omnivory, carnivory, herbivory; migratory, or resident) had its core and unique ASVs (Amplicon Sequence Variants). Diet significantly altered the composition of the birds' gut microbiota, while migration strategy had a small but significant effect (*p* < 0.05). The dispersion in migratory birds was similar to that in resident birds, suggesting that migration does not increase the individual variability of bird gut microbiota. Overall, our findings reveal the associations between the avian gut microbiota and their diet or migration behavior.

## Graphical abstract

This study analyzed the gut microbiota of birds from different ecological types in Nanning, Guangxi, and found that different dietary habits (omnivory, carnivory, and herbivory) and migratory strategies (migratory and resident) significantly altered the gut microbiota of birds, but migration did not increase the inter‐individual variability of gut microbiota. The findings contribute to a deeper understanding of the relationship between the gut microbiota of wild birds and their dietary or migratory behavior.

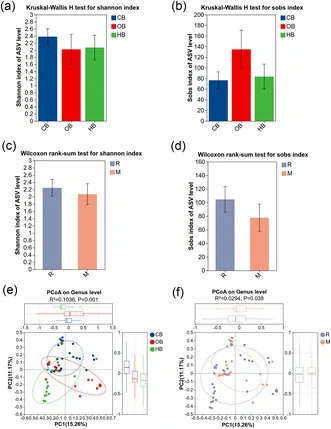

## 1 Introduction

The microbiota is defined as the collection of bacteria in a specific environment (Debré 2016), and the most influential vertebrate symbiotic microbiota exists in the intestine (Adak and Khan 2019). These microbial communities have co‐evolved with their hosts through complex interactions in a mutually beneficial symbiotic relationship that can affect the host's nutrition, physiology, development, and immune system (Sun et al. 2022; Bodawatta et al. 2021). As an important branch of vertebrates, birds occupy diverse ecological niches and play important roles in natural ecosystems. For flight adaptation, wild birds typically have shorter digestive tracts and less intestinal retention (Denbow 2015), and their gut microbiota may be less stable than those of other animals (Hird 2017). Ecological factors such as diet and migration may further affect the composition and diversity of the gut microbiota (Dong et al. 2022).

In avian research, diet is often considered an important regulatory factor that can affect the taxonomic composition, diversity, and function of the gut microbiome (Xiao et al. 2021; Rohrer et al. 2023). Previous studies have shown that bacteria may transfer from prey to predators through trophic networks, thereby affecting the composition of avian gut microbiota (Dion‐Phénix et al. 2021; Laviad‐Shitrit et al. 2017). The critically endangered Siberian Crane ( *Grus leucogeranus* ) has shifted from *Vallisneria* tubers to crops, and the gut microbiota of the two foraging groups was significantly different, indicating that changes in the host's dietary niche promoted changes in gut microbiota (Wang, Wang, et al. 2023). Several studies have found that different dietary habits of birds lead to changes in the diversity of gut microbiota. Among migratory birds wintering in Poyang Lake, omnivorous birds have higher gut microbiota diversity than carnivorous and herbivorous birds (Wang et al. 2022), while another study on birds in Tianjin found that carnivorous birds have the highest gut bacterial diversity (Liu et al. 2024). However, in ecological studies of several waterbirds and forest birds, the richness of plants and invertebrates in bird food was not a significant explanatory variable for fecal microbiota richness (Fablet et al. 2025). The actual food consumption of different individuals may vary greatly, so there is no consistency in the dietary habits and alpha diversity of microbial communities in different studies. In addition, diet is related to the function of the gut microbiota. The gut microbiota of certain bird species has a well‐developed detoxification capability, allowing the hosts to consume toxic food resources. For example, fermentation in the cecum of the western capercaillie ( *Tetrao urogallus* ) is crucial for detoxifying pine needles (Rasmussen and Chua 2023). Diet is consistently linked to various aspects of the gut microbiota, but current research focuses on birds with specific feeding habits; thus, there is a lack of relevant data on the microbiota of different dietary guilds.

The gut microbiome also changes with diet and habitat associated with migration. Migration exposes birds to more diverse and complex environments during their flight, and one way in which animals regulate adaptive responses to environmental changes is through interactions with their gut microbiota, such as increasing the abundance of bacteria that obtain energy from food (Craig 2002; Alberdi et al. 2016). The Blackpoll Warbler ( *Setophaga striata* ) increases the relative abundance of Enterobacteriaceae during migration, which may be related to the metabolic needs of migratory birds (Trevelline et al. 2023). Some studies have also found a trend of decreasing gut microbiota diversity during migration; for example, the microbiota diversity of Nearctic‐Neotropical migratory birds during spring and autumn migration was lower than during breeding periods (Skeen et al. 2023). In addition, the composition of the gut microbiota changed during bird migration. Schmiedová et al. (2023) compared the gut microbiota composition of two temperate migratory bird species, the Garden Warbler ( *Sylvia borin* ) and the Willow Warbler ( *Phylloscopus trochilus* ), with that of resident birds in breeding and non‐breeding areas. The results showed that the gut microbiota of migrating individuals changed, similar to that of resident birds. Migratory birds were exposed to a more diverse new microbiome and pathogens, and engaged in potential microbial exchange with local resident birds (Cohen 2023). Therefore, there is an urgent need to understand the structure and function of gut microbiota across species of wild birds to identify pathways for exploring potential impacts on host birds and human public health. Comparing the gut microbiota of migratory and resident birds can effectively explore the association between migration and changes in the composition and function of the gut microbiota.

Against the backdrop of existing research support, we propose two testable ecological hypotheses. Firstly, we hypothesize that the beta‐diversity of gut microbiota differs among dietary guilds in wild birds, reflecting diet‐associated ecological constraints. Secondly, we hypothesize that migratory strategy is associated with increased inter‐individual variability in gut microbiota composition, consistent with greater environmental exposure and dietary heterogeneity. The microbial composition of migrating Barn Swallow ( *Hirundo rustica* ) and non‐migratory subspecies was different, and migrant microbial communities vary more than those of residents, indicating that migration may further affect the composition and structure of gut microbiota (Turjeman et al. 2020). Therefore, we predict that the community variability within migratory bird populations is greater than that of resident birds (i.e., the Permdisp test of beta‐diversity is significantly higher in migratory bird groups than in resident birds). To verify our hypothesis, we (1) compared the composition and diversity of gut microbial communities among birds from different dietary guilds and migratory statuses; (2) determined the microbial biomarkers for different groups. In addition, we also (3) explored the interaction patterns of microbial communities and functional characteristics of microbiome, as a basis for preliminary exploration of how diet and migration strategies influence the interaction patterns and functions of avian gut microbiota. This study may deepen our understanding of the association between gut microbiota and diet or migration of wild birds.

## 2 Methods

### 2.1 Sample Locations and Sampling

Between March 2024 and July 2025, a total of 61 samples were collected from Nanning Wuxu International Airport, Guangxi, China. Nanning is located in the southern part of Guangxi, south of the Tropic of Cancer, and is an important stopover for migratory birds on the East Asian‐Australasian Flyway (Yuan et al. 2021). The region has a subtropical monsoon climate, with abundant water resources and heavy rainfall, providing excellent habitat for birds. In addition to the diverse resident birds, numerous migratory bird species breed and overwinter in the area every year (Jiang 2021; Ming and Du 2021). There are artificially constructed green spaces at the airport, as well as lush vegetation surrounding it, including trees, farmland, and shrubs, as well as streams, ditches, ponds, and other water bodies that attract birds to settle.

All bird samples from the airport were collected from the bird strike prevention system in accordance with the airport's operating procedures. Airport staff discovered bird carcasses during daily patrols and removed them before sending them to the laboratory, using dry ice for low‐temperature transportation. The bird carcasses were dissected and sampled as soon as they arrived at the laboratory. The samples comprised 25 bird species from five orders and 14 families. Three dietary guilds could be classified: herbivores (*n* = 17), carnivores (*n* = 26), and omnivores (*n* = 18). Among them, carnivorous birds can be further divided into raptors, waterbirds, and insectivorous birds, named CB‐R (*n* = 7), CB‐W (*n* = 7), and CB‐I (*n* = 12), respectively. The dietary guilds information was obtained from ebird (https://ebird.org/prefs). In addition, birds can also be classified by migratory statuses (residents, *n* = 41; and migrants, *n* = 20) (Jiang 2021). To minimize the impact of seasonal variations, all samples of each species were collected in the same season, except for Light‐vented Bulbuls ( *Pycnonotus sinensis* ), which were obtained during the breeding period. Detailed information concerning the birds is provided in Table S1.

Before dissection, we sterilized the dissection tools and collected the birds' cloacae and contents, and then placed the samples in sterile freezing tubes filled with anhydrous ethanol. All samples were placed in the laboratory's −80°C ultra‐low temperature freezer until DNA extraction.

### 2.2 DNA Extraction, PCR Amplification, and Sequencing

The samples were centrifuged, and the supernatant was discarded. The total DNA was extracted from 0.2 g of the cloacal contents using a Soil DNA Extraction Kit (Shanghai Majorbio Bio‐pharm Technology Co. Ltd., Shanghai, China) following the manufacturer's instructions. The integrity of the DNA was detected by 1% agarose gel electrophoresis. Microbiota structural studies were conducted via PCR amplification using the primers 341F [5′‐CCTAYGGGRBGCASCAG‐3′] and 806R [5′‐GGACTACNNGGGTATCTAAT‐3′] to amplify the 16S rRNA gene V3‐V4 region. The PCR reaction mix contained 10 μL of 2 × Pro Taq, 0.8 μL of each primer, 10 ng/μL DNA template, and ddH_2_O to a final volume of 20 μL.

The PCR cycling conditions were as follows: an initial denaturation at 95°C for 3 min, followed by 29 cycles of 95°C for 30 s, 53°C for 30 s, and 72°C for 45 s, and a final elongation step at 72°C for 10 min. For the 3 PCR negative controls, DNA was replaced with sterile water, and no product was detected. We performed three technical replicates for each sample, and the PCR products from the three replicates were combined. The amplification products were confirmed by 2% agarose gel electrophoresis, and an AxyPrep DNA Gel Extraction Kit (AXYGEN, USA) was used to cut and recover the PCR products. Based on the preliminary quantitative results from the electrophoresis, the PCR products were analyzed using QuantiFluor‐ST blue fluorescence quantification system (Promega, USA) for detection and quantification. A sequencing library was constructed using the TruSeq DNA Sample Prep Kit (Illumina, USA) according to the manufacturer's instructions. High‐throughput sequencing analysis of 16S rRNA gene amplicons was performed on the Illumina MiSeq platform by Shanghai Majorbio Bio‐pharm Technology Co. Ltd. (Shanghai, China).

### 2.3 Sequencing Data Processing

Based on the sequencing quality, fastp v0.23.4 (https://github.com/OpenGene/fastp) was used to perform quality control and filtering on the paired‐end reads. At the same time, FLASH v1.2.11 was used to concatenate overlapping regions between paired reads to obtain optimized data after quality control. The DADA2 plugin in QIIME2 was used to denoise the sequencing reads. The denoising steps include filtering out noise and correcting sequence errors, removing chimeras, single sequences, and duplicate sequences to obtain high‐resolution ASVs for subsequent analysis (Data S1). ASVs that matched mitochondrial and chloroplast sequences were removed from the dataset. The Naive Bayes classifier and the Silva 138.2 database were used for taxonomic annotation of ASV representative sequences, with a confidence level of 70%.

### 2.4 Statistical Analysis

Mothur v1.30.2 (https://mothur.org/wiki/calculators/) was used to calculate the microbial alpha diversity index at different sequencing depths to construct a rarefaction curve and calculate alpha diversity indices (including Shannon and Sobs indices). A curve chart was created with R v3.3.1 (https://www.r‐project.org/). Corresponding statistical methods were used to test the differences in alpha diversity between groups: For different dietary guilds, the Kruskal‐Wallis rank‐sum test was used; for different migratory statuses, the Wilcoxon rank‐sum test (two‐tailed) was used for analysis. Beta diversity analyses were performed using the Bray‐Curtis, weighted UniFrac, and unweighted UniFrac distances (Lozupone et al. 2011), with the analyses performed in R v3.3.1. We used Principal Coordinate Analysis (PCoA) to visualize the differences in bacterial genera between groups. Significant differences were tested using PERMANOVA to assess differences in microbial community composition among different ecological types of birds, and evaluated the differences in inter‐individual variability within the group using Permdisp test. Venn diagrams were used to visualize the number of shared and unique ASVs in each group (Shade and Handelsman 2012).

Linear discriminant analysis effect size (LEfSe) (Segata et al. 2011) was used to identify the biomarker species distinguishing two or more biological conditions (or taxa). The specific steps were as follows: For different dietary guilds, first, use the Kruskal‐Wallis rank‐sum test to identify features and groups with significant differences in abundance. Then, the Wilcoxon rank‐sum test was used to determine the specific groups with significant differences. Finally, LEfSe used linear discriminant analysis (LDA) to estimate the effect size of individual taxa (LDA score ≥ 4.0, *p* < 0.05). The Wilcoxon rank‐sum test was used to screen taxonomic groups for birds with different migratory statuses, and then LDA analysis was used (Ijaz et al. 2018).

Network analysis based on correlation has been widely applied to explore microbial relationships (Faust and Raes 2012). To predict potential associations between the core microbiome (the top 50 bacterial genera of relative abundance), the correlation networks were constructed. The analysis used the Networkx (version 1.11) toolkit and Spearman correlation to calculate the node degree distribution and topological features such as node connectivity (degree) and central coefficient values (closeness centrality, betweenness centrality, and degree centrality) of the network to obtain the bacterial co‐occurrence patterns. The larger these values of a node, the more important it is in the network. A significance threshold of *p* < 0.05 and an absolute value of a correlation coefficient greater than 0.7 indicated a significant association. The network diagrams were visualized using Gephi 0.9.2.

PICRUSt2 (http://huttenhower.sph.harvard.edu/galaxy, v2.2.0‐b) was used to predict the functional potential of bacterial communities (Langille et al. 2013). The metabolism and function of the gut microbiome in birds were annotated based on the Kyoto Encyclopedia of Genes and Genomes (KEGG) database (Kanehisa et al. 2014). The Kruskal‐Wallis rank‐sum test was used for significant differences in different dietary guilds, and the Dunn test was used for post hoc analysis; use the Wilcoxon rank‐sum test for different migratory statuses. All statistical tests were conducted using the Benjamin Hochberg method to control for false discovery rate (FDR). For all statistical tests, a *p*‐value < 0.05 was considered statistically significant.

## 3 Results

### 3.1 Bird Gut Microbiota Alpha and Beta Diversity

A total of 9,261,666 raw reads were obtained through sequencing. After quality control splicing, 3,859,417 optimized sequences were recovered. After denoising, 3,312,259 high‐quality reads and 8479 ASVs were obtained. A total of 3684 ASVs were obtained after removing chloroplast and mitochondrial sequences (Table S2). The rarefaction curves constructed based on the Shannon index indicated that the sample sequencing depth was sufficient to reflect the abundance of most microbial communities in each sample (Figure S1).

**FIGURE 1:**
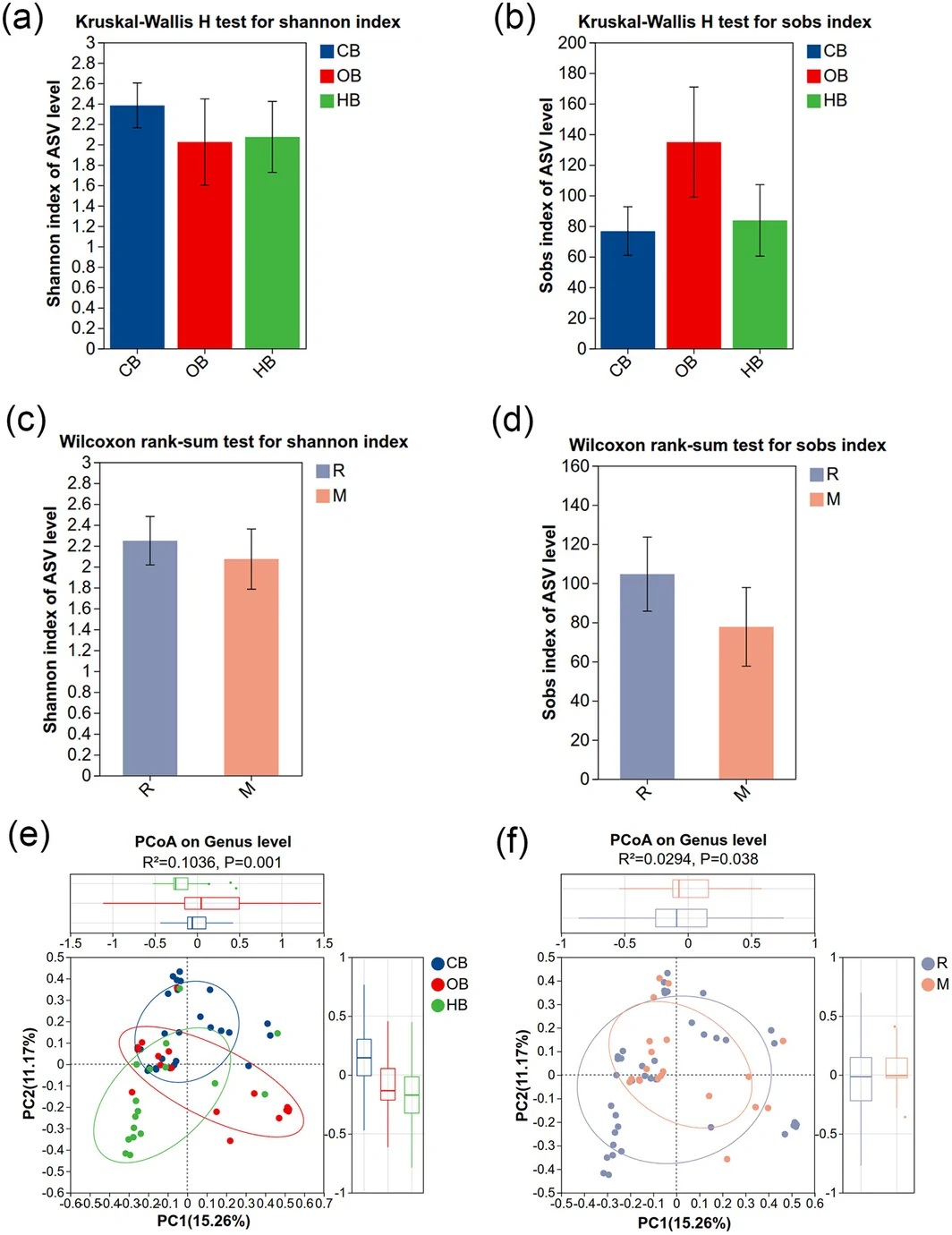
Gut microbiota diversity among different dietary and migratory bird groups. (a) and (b) represent the Shannon and Sobs indices for each diet group. (c) and (d) represent the Shannon and Sobs indices for the migratory groups. The error bar represents the standard error (SE). PCoA among diet groups (e) and migratory groups (f). CB, carnivore birds; HB, herbivore birds; M, migrant birds; OB, omnivore birds; R, resident birds.

We used the Sobs and Shannon indices based on the ASVs to characterize alpha diversity. The results showed that the Sobs indices of the omnivore birds (OB) were higher than those of the herbivore birds (HB) and carnivore birds (CB); the Shannon index of the CB group was higher than that of the other two groups, although there were no significant differences (*p* > 0.05; Figure 1a,b). Similarly, there were no significant differences between the migration groups, and the Sobs and Shannon indices of the resident birds (R) were slightly higher than those of the migrant birds (M) (*p* > 0.05; Figure 1c,d).

Principal coordinates analysis (PCoA) based on the Bray‐Curtis distance showed that microbial community composition varied among dietary and migratory groups (*p* < 0.05; Figure 1e,f). Diet and migration status can explain 10.36% and 2.94% of microbial variation, respectively (*R* ^2^ = 0.1036; *R* ^2^ = 0.0294). There was no significant difference in Permdisp test among different migration status groups (*p* = 0.907), indicating that the differences between groups were not due to differences in intra‐group dispersion. The variability of migratory and resident birds was similar at the individual level. The differences in beta‐diversity were also significant using the unweighted UniFrac and weighted UniFrac distances, but only among dietary groups (*p* < 0.01; Figure S2).

### 3.2 Microbiota Composition

**FIGURE 2:**
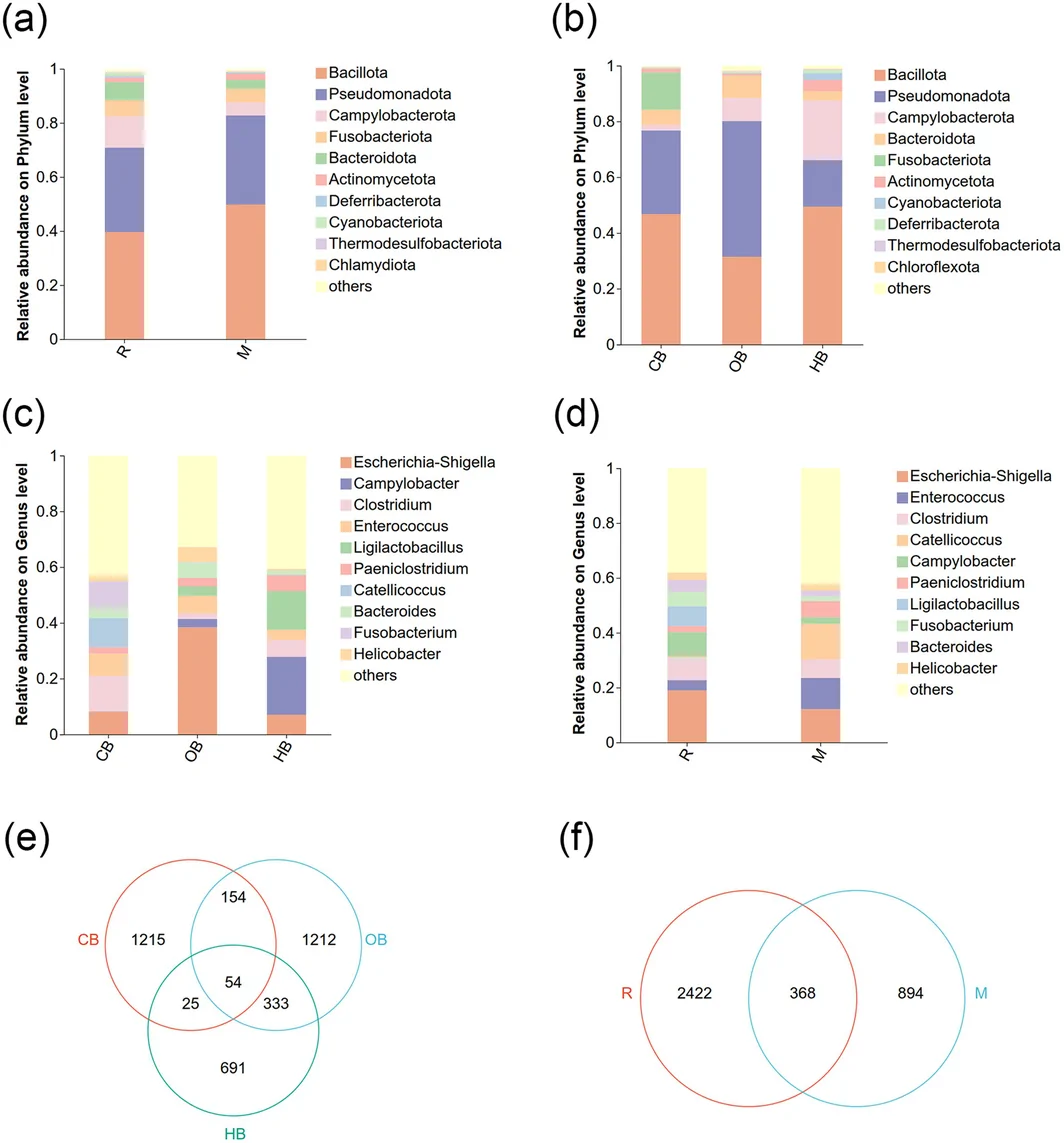
Comparison of the composition of the microbiome among different ecological types. Relative abundance at the phylum level among different dietary (a) and migratory statuses (b), and at the genus level for dietary (c) and migratory statuses (d). The Venn diagram of the ASVs among different dietary (e) and migratory (f) groups. CB, carnivore birds; HB, herbivore birds; M, migrant birds; OB, omnivore birds; R, resident birds.

The ASVs were assigned to 31 phyla, 71 classes, 171 orders, 319 families, 754 genera, and 1085 species. We analyzed the bacterial composition of the bird samples at the phylum and genus levels (Figure 2a,b). The five most abundant bacterial phyla in all samples included Bacillota (42.99%), Pseudomonadota (31.87%), Campylobacterota (9.32%), Fusobacteriota (5.73%), and Bacteroidota (5.53%), collectively accounting for 95.44% of the total sequences. The bacterial phylum with the highest abundance in OB was Pseudomonadota (48.69%), followed by Bacillota (31.45%), Campylobacterota (8.33%), and Bacteroidota (7.87%). For the CB and HB groups, the most abundant phylum was Bacillota (CB, 46.76%; HB, 49.44%). The dominant phyla in the HB group also included Campylobacterota (21.44%) and Pseudomonadota (16.65%), while in the CB group, these were Pseudomonadota (29.97%) and Fusobacteriota (13.31%). Notably, Fusobacteria was only as a dominant phylum in the CB group, with extremely low levels in the other groups (Figure 2a). Furthermore, both resident and migrant birds shared the same dominant bacterial phyla but with different proportions (Figure 2b), including Bacillota (M, 49.84%; R, 39.65%), Pseudomonadota (M, 32.94%; R, 31.21%), Campylobacterota (M, 4.79%; R, 11.54%), Bacteroidota (M, 3.19%; R, 6.67%), and Fusobacteriota (M, 5.12%; R, 6.03%).

The dominant genera were *Escherichia‐Shigella* (Pseudomonadota; 16.74%), *Clostridium* (Bacillota; 7.78%), *Campylobacter* (Campylobacterota; 6.69%), *Enterococcus* (Bacillota; 6.25%), and *Ligilactobacillus* (Bacillota; 4.83%). *Escherichia‐Shigella* accounted for 38.42% in OB, but only 8.10% and 7.03% in CB and HB, respectively. The top three genera in the CB group were *Clostridium* (12.79%), *Catellicoccus* (Bacillota; 10.40%), and *Fusobacterium* (Fusobacteriota; 9.58%). OB was dominated by *Enterococcus* (6.27%), *Bacteroides* (5.43%), and *Helicobacter* (5.38%), while *Campylobacter*, *Ligilactobacillus* (Bacillota), *Clostridium*, and *Paeniclostridium* were the dominant microbial genera in HB, accounting for 20.49%, 13.82%, 6.13%, and 5.75%, respectively (Figure 2c). For the R group, the bacterial genera with the highest abundance were *Escherichia‐Shigella* (18.97%), followed by *Campylobacter* (8.82%), *Clostridium* (8.30%), and *Ligilactobacillus* (7.18%). The top five genera in the M group were *Catellicoccus* (12.96%), *Escherichia‐Shigella* (12.18%), *Enterococcus* (11.41%), *Clostridium* (6.72%), and *Paeniclostridium* (5.92%) (Figure 2d).

The Venn diagram shows the number of ASVs shared and unique to birds with different feeding habits and migratory statuses. A total of 387 (15.67%) ASVs were shared by the OB and HB groups, followed by 208 (6.95%) ASVs between OB and CB, and only 79 (3.20%) shared ASVs between the HB and CB groups. There were 54 (1.47%) ASVs shared among three diet groups (Figure 2e). In addition, resident and migrant birds shared 368 ASVs, accounting for 29.16% of the M group and 13.19% of the R group, respectively; the respective percentages of unique ASVs belonging to the M and R groups were 70.84% and 86.81% (Figure 2f).

### 3.3 Differences in Microbiota Composition

**FIGURE 3:**
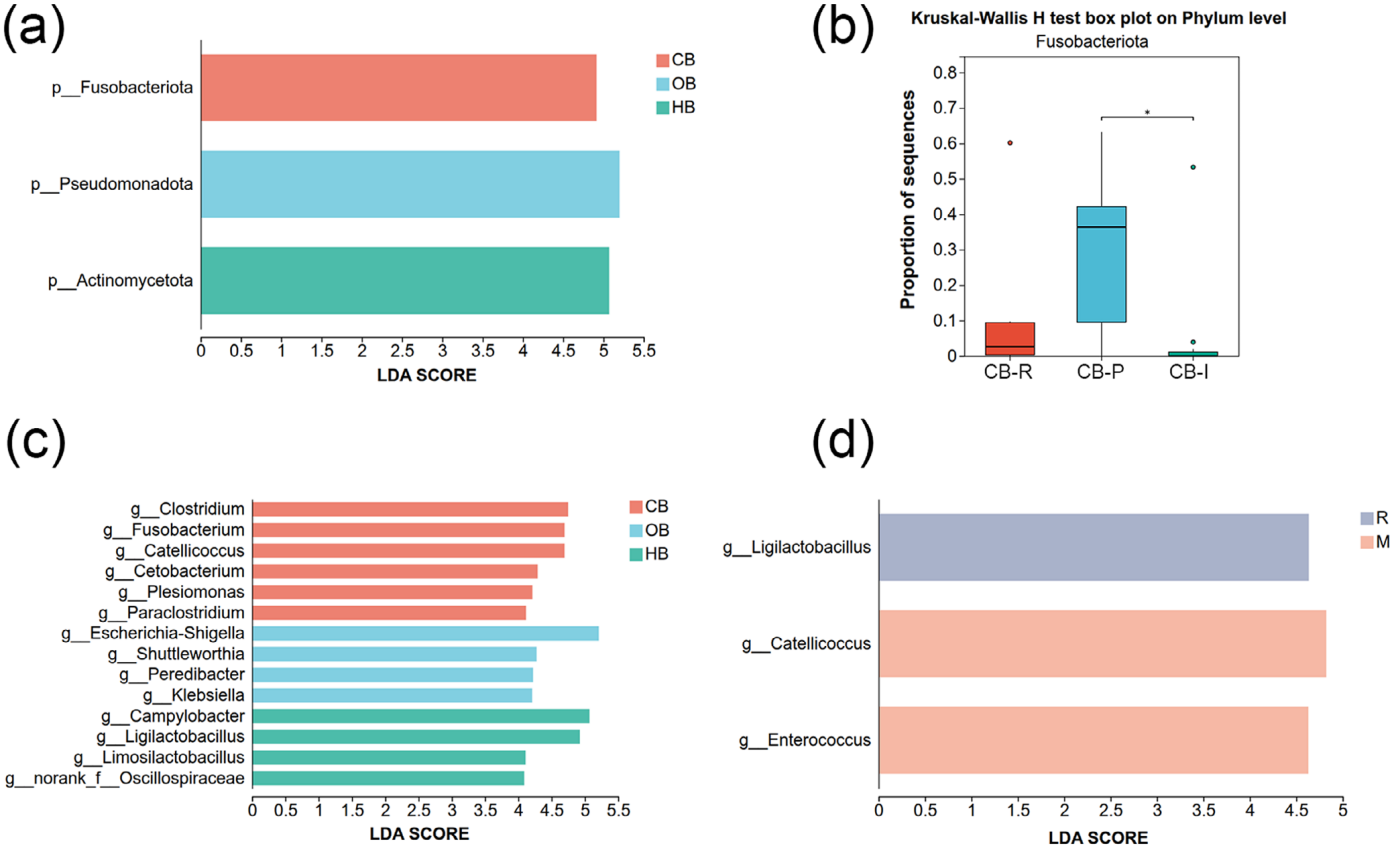
Species difference analysis showing the biomarkers among different dietary and migratory groups. LEfSe analysis with different dietary habits at the phylum level (a). Significance test of Fusobacteriota between CB‐R, CB‐W, and CB‐I (b) *p < 0.05. LEfSe analysis with different dietary (c) and migratory status (d) at the genus level. LDA score ≥ 4.0 and *p* < 0.05. CB, carnivore birds; CB‐I, insectivores; CB‐R, raptors; CB‐W, waterbirds; HB, herbivore birds; M, migrant birds; OB, omnivore birds; R, resident birds.

LEfSe was used to characterize the different microbial communities among the ecological types of birds (Figure 3). The results showed significant enrichment of bacterial phyla in all three dietary groups, with Pseudomonadota in the OB group, Actinomycetota in the HB group, and Fusobacteriota in the CB group (Figure 3a). In the carnivorous birds, the high abundance of Fusobacteriota was largely contributed by waterbirds (CB‐W), and the abundance of this bacterial phylum in CB‐W was significantly higher than that in insectivores (CB‐I) (Figure 3b). At the genus level, we observed biomarkers for 14 taxa, of which six taxa were significantly enriched in the CB group (*Catellicoccus*, *Fusobacterium*, *Clostridium*, *Cetobacterium*, *Plesiomonas*, and *Paraclostridium*). The HB group exhibited enrichment in *Campylobacter*, *Ligilactobacillus*, *Limosilactobacillus*, and g__norank_f__*Oscillospiraceae*, while the enriched taxa in the OB group were *Klebsiella*, *Escherichia‐Shigella*, *Shuttleworthia*, and *Peredibacter* (Figure 3c). In addition, there were no differences at the phylum level between the M and R groups, but there were three significantly different taxonomic groups at the genus level, among which *Ligilactobacillus* was enriched in R, and the gut of migratory birds was rich in *Catellicoccus* and *Enterococcus* (Figure 3d).

### 3.4 Bacterial Co‐Occurrence Patterns

Co‐occurrence networks were constructed to visualize the patterns of statistical associations among bacteria. There were positive correlations between all bacterial genera. The co‐occurrence network analysis of different feeding birds showed that the OB group had 37 nodes, the largest number of edges (242), and the highest average clustering (0.79), indicating that the bacterial community in the OB group was the most closely connected and the interaction was the most complex; the network in HB consisted of 40 nodes and 138 edges; the average clustering was 0.77, indicating that the bacterial community connection density of the HB group was lower than the OB group, while the number of network nodes (27) and the number of edges (42) of the CB group were the least among the three groups, and the average clustering coefficient was the lowest (0.45), indicating that the network structure was the simplest among the three dietary groups. The analysis in different migration statuses showed that the number of network nodes in the R group (28) and the M group (26) were close, and the R group had a higher number of edges (R, 63; M, 38), and the average clustering was slightly higher than that in the M group (R, 0.52; M, 0.44), indicating that the complexity of the relationship between the M group bacterial communities and the R group was comparable, but the connectivity and interaction intensity were slightly higher than that in the M group.

**FIGURE 4:**
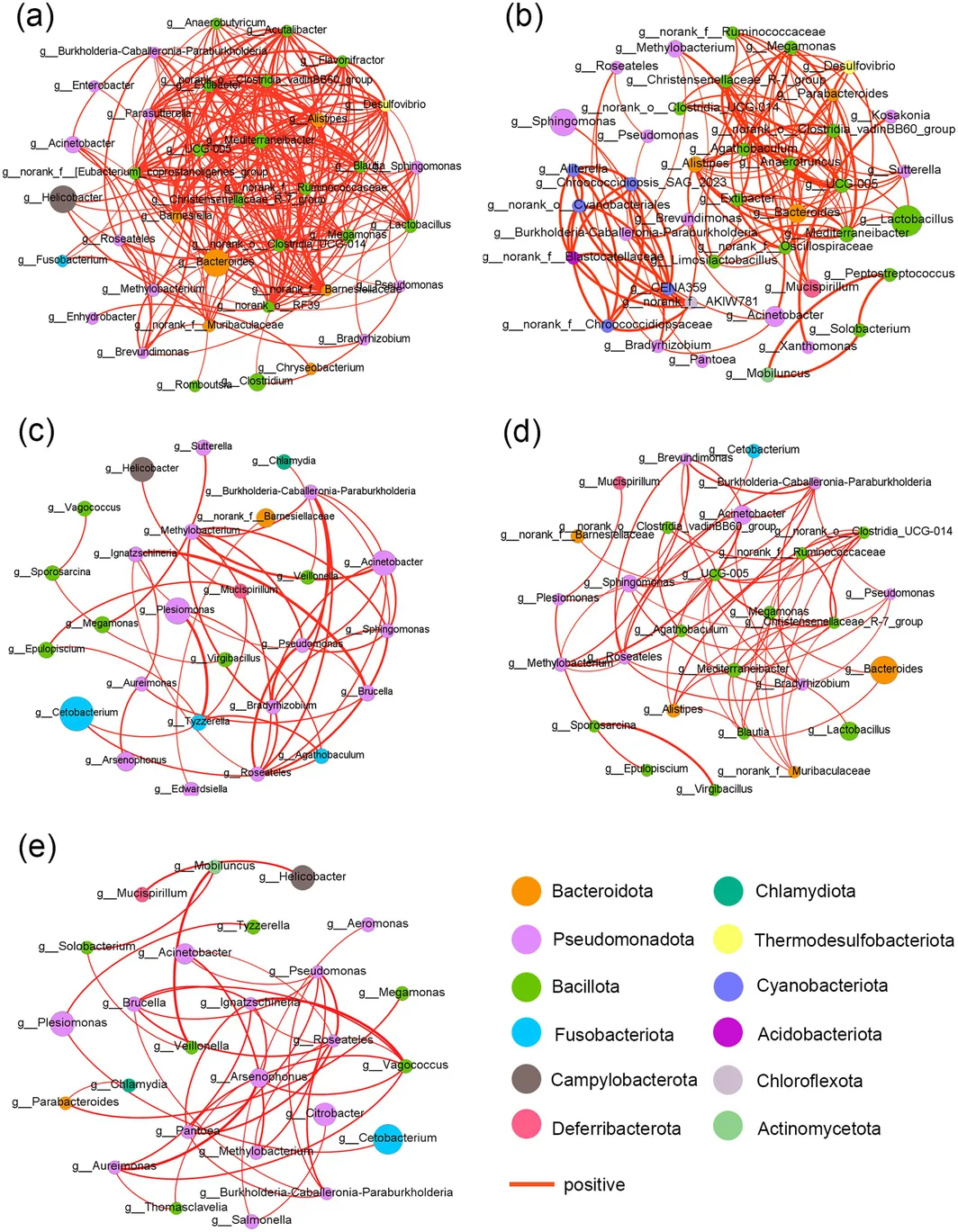
Co‐occurrence networks of gut microbiota in OB (a), HB (b), CB (c), R (d), and M (e) groups with Spearman correlation coefficients > |0.7| and *p* < 0.05. Each node represents a genus. Node colors represent the associated phylum for each genus, and the node size is proportional to abundance. The red edges indicate positive correlations. The edge width indicates the strength of correlation. CB, carnivore birds; HB, herbivore birds; M, migrant birds; OB, omnivore birds; R, resident birds.

In the network of OB group, *Alistipes* had the highest degree number of 24. Two bacterial genera with relative abundance ranking in the top ten in the OB group, *Bacteroides* (5.43%) and *Helicobacter* (5.38%) were connected to 23 and 5 taxa, respectively (Figure 4a). *Sphingomonas* (3.05%) was the bacterial genus with the higher abundance in the network of HB group, which was connected to *Bradyrhizobium* and *Burkholderia*‐*Caballeronia*‐*Paraburkholderia* (Figure 4b). *Pseudomonas* has the highest number of connections in the network of CB group, and was also the most important genus in the network. The high relative abundances of genera, such as *Cetobacterium* (3.73%), were associated with *Agathobaculum* and *Tyzzerella* (Figure 4c). The bacterial genera with the highest node connectivity in the R group were *UCG‐005*, *norank_o__Clostridia_UCG‐014*, and *Mediterraneibacter*, all connected to nine nodes, and *UCG‐005* was the most important genus in the network (Figure 4d). Roseateles was the bacterial genus with the highest connectivity in the network of the M group, which is connected with 8 bacterial genera (Figure 4e).

### 3.5 Predictions of the Function of the Gut Microbiota

**FIGURE 5:**
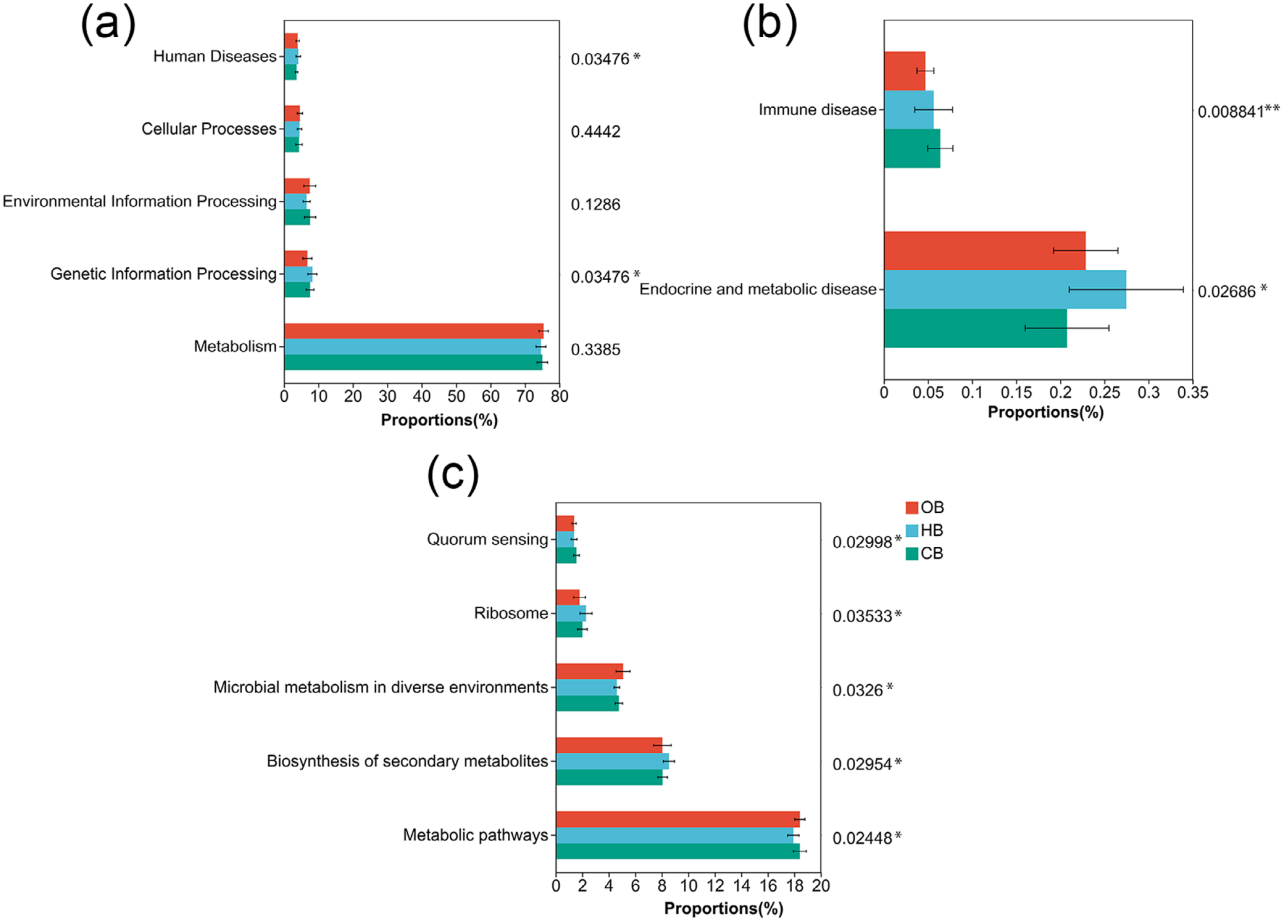
Comparison of microbial functions among different dietary groups. Functional differences among different diets in the KEGG level I pathways (a), KEGG level II pathways (b), and KEGG level III pathways (c) based on the Kruskal‐Wallis rank‐sum test. CB, carnivore birds; HB, herbivore birds; OB, omnivore birds. *p < 0.05, **p < 0.01.

Potential functions of all ASVs were annotated by PICRUSt2. Considering the constraints inherent to 16S‐based taxonomic inference, all resulting functional predictions are hypothesis‐generating rather than empirically verified conclusions. We did not find significant differences in KEGG functional pathways by migratory statuses (*p* > 0.05); thus, only detailed analysis between dietary groups was conducted. The level I pathways were clustered into metabolism, genetic information processing, environmental information processing, cellular processes, human diseases, and organismal systems. Among these, metabolism was the dominant functional pathway, accounting for more than 70% (Figure 5a). Genetic information processing, environmental information processing, and human diseases displayed significant differences among the three dietary groups (*p* < 0.05). It is worth noting that the human disease pathway in the HB group was significantly higher (*p* < 0.05). The level II pathways of microbial functions were dominated by global and overview maps. Among the level II pathways, immune disease was enriched in the CB group (*p* < 0.01), while endocrine and metabolic disease of HB was significantly higher than in the other two groups (*p* < 0.05) (Figure 5b). In the level III pathways, a total of 134 (406 in total) functional pathways showed significant differences (*p* < 0.05). Metabolic pathways, biosynthesis of secondary metabolites, microbial metabolism in diverse environments, ribosome, and quorum sensing were the most enriched, and there were significant differences between the dietary groups (*p* < 0.05) (Figure 5c). Metabolic pathways were significantly higher in OB and CB. Microbial metabolism in diverse environments was enriched in OB, whereas biosynthesis of secondary metabolites was significantly higher in HB.

## 4 Discussion

In this study, the most abundant bacterial phyla in the gut microbiota of all birds were Bacillota, Pseudomonadota, Campylobacterota, and Bacteroidota. These are typical bacterial phyla in Aves (Grond et al. 2018; Boukerb et al. 2021) and may play important roles in the food digestion, nutrient absorption, metabolism, and immunity of wild birds. For example, Bacillota and Bacteroidota are able to decompose complex carbohydrates, polysaccharides, cellulose, and other polymers, thereby producing beneficial metabolites for the host, such as short‐chain fatty acids (Doifode et al. 2021). Meanwhile, these two bacterial phyla also contribute to effective heat absorption and defense against intestinal pathogens (He et al. 2019). Significant differences in microbial communities were identified in different dietary guilds. At the phylum level, Actinomycetota was significantly enriched in the HB group, Fusobacteriota was enriched in the CB group, and Pseudomonas was a biomarker for the OB group (Figure 3a). Most Actinomycetota are widely distributed in water and soil, and they are related to the intake of host fiber (Zhao, Liu, et al. 2022). This was consistent with the HB group's dietary preferences for grains. Compared to herbivorous and omnivorous birds, the cloacal microbiota of carnivorous birds had a higher abundance of Fusobacteriota. Notably, although they belong to the carnivorous group, fish‐eating waterbirds (CB‐W) such as Gray Heron ( *Ardea cinerea* ), Green‐backed Heron ( *Butorides striata* ), and Schrenck's Bittern ( *Ixobrychus eurhythmus* ) had a higher proportion of Fusobacteria, which was almost absent in the insectivorous birds (CB‐I) such as Richard's Pipit ( *Anthus richardi* ), Hair‐crested Drongo ( *Dicrurus hottentottus* ), and Red‐rumped Swallow ( *Lanius cristatus* ). Our research was in consistency with those of previous studies, the prevalence of Fusobacteriota was observed in Relict Gulls ( *Ichthyaetus relictus* ), Gentoo Penguins ( *Pygoscelis papua* ), and shorebirds (Wu et al. 2024; Kaczvinsky et al. 2024; Zhang et al. 2021). Zhu et al. (2021) found that Crested Ibis ( *Nipponia nippon* ) chicks contained Fusobacteriota in their intestines after consuming fresh loaches, and this phylum was also common in various fish species (Kanika et al. 2025). Therefore, we speculate that the high proportion of Fusobacteriota in waterbirds may be derived from fish ingestion. It is necessary to collect food samples from birds for comparative analysis to verify this viewpoint. The phylum Pseudomonas, which dominated in the OB group, was abundant in natural environments and is often influenced by humans. The phylum contains a large number of opportunistic pathogens (Leão et al. 2023). Pseudomonas and Fusobacteriota are capable of anaerobically degrading various purines, including uric acid, as carbon and energy sources (Kasahara et al. 2023). At the genus level, *Campylobacter* and *Ligilactobacillus* were enriched in herbivorous birds and were dominant in the guts of both vegetarians and granivores (García‐Amado et al. 2018; Yi et al. 2023). The herbivorous birds in our study feed on paddy rice, and starch is the main nutrient in rice. These complex polysaccharides are difficult for vertebrates to digest, and may require specific bacterial symbionts to mediate the breakdown of complex carbohydrates. In carnivorous birds, most of the identified bacterial genera, including *Catellicoccus, Plesiomonas*, and *Cetobacterium* are involved in nutrient transport, lipid metabolism, and bile acid hydrolysis (Góngora et al. 2021; Wang et al. 2021; Zhao, Mao, et al. 2022). Additionally, *Clostridium* plays a major role in the degradation of non‐starch polysaccharides. *Enterococcus* can decompose proteins and esters (Du et al. 2020; García‐Solache and Rice 2019), which may help carnivorous birds digest food resources such as insects and mice. The above results indicate that different dietary habits may promote the differentiation of gut microbiota. There were three biomarkers in the gut microbiota of resident and migratory birds (Figure 3d). Among them, *Catellicoccus* and *Enterococcus*, which were significantly enriched in migratory birds, are related to lipid metabolism. According to reports, some metabolites produced by lipid metabolism, such as glucose, fatty acids, and lactate, play a crucial role in active migration, primarily through lipid metabolism (Butler 2016; Guglielmo 2010). Whether these microbial communities have functional effects on the host's physiology or metabolism (e.g., aiding in the digestion of specific foods) requires further experimental verification.

Although the differences were not statistically significant, in our survey, omnivorous birds had the highest species richness in gut microbiota, while carnivorous birds had the highest Shannon diversity, and resident birds had higher alpha diversity than migratory birds. The CB group showed a trend of higher community diversity and a more balanced distribution of species, which is consistent with the research results of Liu et al. (2024); a trend of reduced microbial diversity has been observed in migratory birds, which is consistent with the hypothesis put forward by Skeen et al. that the migration process limits gut microbial diversity (Skeen et al. 2023).

We found that birds with different dietary guilds have significantly different microbial communities. Regardless of using Bray‐Curtis distance, unweighted UniFrac distance, or weighted UniFrac distance, there were significant differences in beta‐diversity among different dietary guilds (Figure 1e and Figure S2), which supports our first hypothesis (gut microbiota beta‐diversity differs among dietary guilds in wild birds). Different food sources shaped the characteristics of the gut microbiota, and diet mainly drives the differentiation of microbial communities by altering the relative abundance and phylogenetic lineage structure of dominant communities (Lozupone et al. 2011). For different migration status groups, significant differences were found in beta‐diversity analysis based on Bray‐Curtis distance (Figure 1f), indicating that migration strategies significantly affect the relative abundance of dominant microbiome in birds, even though this effect only explain 2.94% of microbial variation (PERMANOVA, *R* ^2^ = 0.0294, *p* = 0.038). By comparing the intra‐group dispersion of resident and migratory bird populations, we did not find significant differences in community variability, which contradicted our second hypothesis that community variability within migratory bird populations is greater than that of resident birds. Previous studies have shown that the gut microbiota of migratory birds undergoes rapid changes, ingesting different food and environment related microorganisms at different stopover habitats, thereby increasing the diversity of microbial communities. (Grond et al. 2018; Wu et al. 2018; Włodarczyk et al. 2024). However, the absence of increased variability in migratory birds was observed in this study, which may suggest that exposure to diverse environments does not necessarily translate into greater microbiota heterogeneity at the scale captured by fecal sampling. This view is supported by the fact that the variability among migrating Canada Geese ( *Branta canadensis* ) were no higher than that among resident individuals and even showed a trend toward lower variability (Obrochta et al. 2022). Another possible explanation is that host‐related constraints (e.g., physiological filtering or microbiota resilience) limit the extent of such variability (Mallott and Amato 2021; Risely et al. 2017; Kreisinger et al. 2017). For example, the gut microbiota of Red‐necked Stint ( *Calidris ruficollis* ) exhibits largely resistant against environmental microbe incursions (Risely et al. 2017). Although the effect size of temporal stability was low at the whole‐community level in migratory barn swallows, an adult fecal microbiota subset with relative abundances exhibiting significant temporal consistency was identified, possibly inducing long‐term effects on the host phenotype (Kreisinger et al. 2017). In addition, both migratory and resident exhibit significant inter‐individual variation (Figure 1). This phenomenon can be explained by the characteristics of the fecal samples. On the one hand, the inherent heterogeneity is influenced by internal factors such as individual digestive status and physiological stress, resulting in natural inter‐individual fluctuations in microbial community structure (Lewis et al. 2016). On the other hand, fecal samples are of mixed origin, containing not only the host's indigenous gut microbiota but also being potentially contaminated with environmental exogenous microbial communities or potential pollutants, thereby further amplifying community differences between individuals (Fablet et al. 2025; Skeen et al. 2023).

As an exploratory analytical framework, co‐occurrence network analysis quantifies statistically significant inter‐taxon associations within microbial communities, which encompassing both cooperative and competitive interactions (Deng et al. 2012). The network results indicate that positive connections between different ecological types of bird microbiota predominate. The clustering coefficient is commonly used to describe the hierarchical properties of a network, indicating the degree of connection between a node and its neighbors (Deng et al. 2012). The average clustering coefficients of the OB group was the highest, and the average clustering coefficient of the HB and OB groups were significantly higher than that of the CB group, suggesting the closest relationship among bacteria in omnivorous birds. This is consistent with the research findings of Wang et al. (2022) on migratory birds with different feeding habits. Compared with omnivorous birds, herbivorous and carnivorous birds have reduced microbial network complexity. However, migration did not affect the overall connectivity of the microbial network structure. The most important bacterial genus in the M network was *Roseateles*, which includes strains that degrade chitin, and are associated with other bacteria with various metabolic functions (Cai et al. 2025). *Alistipes*, as the bacterial genus with the most node associations in the OB group, is closely related to *Ruminococcaceae* and *Christensenellaceae*_R‐7, bacteria that can decompose fiber, as well as Bacteroide, which has the ability to utilize carbohydrates and amino acids (Li et al. 2025; Facimoto et al. 2025; Flint and Duncan 2014). In addition, some opportunistic pathogens such as *Clostridia_UCG‐014, Mediterraneibacter*, and *Helicobacter*, dominated the connectivity of the bird networks across ecological types (Wang, Jia, et al. 2023; Mingolelli et al. 2025; Ryan and Pembroke 2018). Notably, co‐occurrence networks based on correlation reflect statistical associations rather than validated biological interactions, serving only as a preliminary basis for exploring patterns of microbial interactions.

The gut microbiota of wild birds exhibited high levels of metabolic activity (Figure 5). Microbial metabolic pathways, such as carbohydrate, amino acid, and purine metabolism, accounted for the majority of the top 30 predicted pathways at level III. Although significant differences in gut microbiota composition existed among birds of different ecological types, their predicted community functional characteristics were highly similar. Functional divergence was only observed in partial pathways among dietary groups, indicating high functional redundancy in avian gut microbiota. Recent studies have shown that migration strategies often change the composition of the microbiome, without necessarily leading to stable functional or adaptive restructuring of the microbiome (Wu et al. 2018; Włodarczyk et al. 2024). In contrast, dietary research has shown that the function and metabolic characteristics of gut microbiota are influenced by diets, and even the function of the same microbiota may vary slightly under different diets (Xiao et al. 2021; De Filippis et al. 2019). However, it should be noted that the primer selectivity of 16S rRNA gene amplicon sequencing may lead to potential bias in taxonomic units, and the measurement of functional genes is also uncertain (Fouhy et al. 2016; Callahan et al. 2019). Therefore, the functional predictions based on 16S data in this study should be regarded as hypothetical rather than validated conclusions. Future dataset analysis based on metagenomics can help solve the problem of how ecological factors such as diet regulate microbial function and classification (Youngblut et al. 2019).

It is worth noting that there were some opportunistic pathogenic microbiome in the gut microbiota of birds. For example, the HB group contained a considerable abundance of *Campylobacter* (20.49%), and *Escherichia‐Shigella* was dominant in all bird taxa. Wild birds are natural hosts of *Campylobacter*, but this genus is the main cause of human foodborne gastroenteritis. There is evidence suggesting that it can spread to poultry and the larger environment (Tawakol et al. 2023). The increase in *Escherichia‐Shigella* induced disturbance of the intestinal microbiome aggravated infection and damaged the gut (Wang, He, et al. 2023). In our study, these pathogens occupy an important position in the microbial network and can interact with other bacteria (Figure 4). In short, as carriers of opportunistic pathogens, wild birds' feces may contaminate the environment and infect humans. Thus, it is necessary to accurately identify pathogens that inhabit wild birds and search for strategies to reduce pathogens and prevent related diseases. It cannot be ignored that we have not isolated and tested the pathogenicity of bird gut microbiota, so the inference that bird feces may infect humans and other animals should be treated with caution. On the other hand, our research suggests that the gut microbiota of birds was influenced by dietary habits and migration strategies; however, it is largely unclear how this change affects host health, especially in terms of potential microbial dysbiosis (Fablet et al. 2025). Several conceptual syntheses and comparative studies highlight the dominant roles of ecological context, host diet, and plasticity in shaping host‐associated microbiota, but cannot infer mechanisms and adaptations (Grond et al. 2018; Youngblut et al. 2019; Capunitan et al. 2020). Further research is needed to explore the relationship between gut microbiota and host adaptation and health.

In summary, we comprehensively analyzed the gut microbiota of birds with different ecological types using 16S amplicon sequencing technology. However, several limitations of this study should be noted. First, bird samples were collected randomly, and some species had only one replicate, which reduced the generalizability of our findings. Second, the opportunistic sampling methods, which were known to reflect a composite signal influenced not only by host‐associated gut communities but also by environmental exposure, stochasticity, and potential contamination more generally, the results could be framed as reflecting ecological patterns filtered through sampling constraints, rather than direct representations of gut microbiota structure (Fablet et al. 2025). Consistently, the relatively weak effect of migration on gut microbiota (PERMANOVA, *R* ^2^ = 0.0294, *p* = 0.038) further reflects the limitations of this sampling method in capturing specific ecological signals. Third, dietary categorization of sampled birds was assigned based on published literature instead of refined dietary analysis at the individual level, which may neglect the influences from plant and invertebrate prey. Methodologically, the 16S amplicon sequencing used in this study permits preliminary functional prediction of gut microbiota, resulting functional inferences remain hypothesis‐generating rather than definitive evidence (Fouhy et al. 2016; Callahan et al. 2019). In the future, further work is needed to quantify the correlation between the dietary structure and microbial community structure of birds, which can be achieved by analyzing the relative nutritional composition of different diets and determining dietary diversity based on DNA metabarcoding of fecal samples (Fablet et al. 2025; Wang, Long, et al. 2023). In addition, metagenomics will enable more accurate characterization of microbial functions, particularly those of pathogens. Isolation and cultivation of fecal microbes are also needed to explore their pathogenicity and infectivity (Youngblut et al. 2019).

## 5 Conclusions

In this study, opportunistic sampling methods of birds of different ecological types were conducted in Nanning, Guangxi (a key stopover habitat on the East Asian‐Australasian Flyway), indicating that different dietary habits (omnivory, carnivory, and herbivory) and migratory strategies (migratory and resident) significantly altered the gut microbiota of birds. Migration did not increase the inter‐individual variability of gut microbiota, suggesting that diverse environmental exposure does not necessarily enhance microbial heterogeneity in fecal samples, or host‐related constraints limit the extent of microbial variation. In summary, our study emphasizes how the gut microbiota of birds responds to dietary and migratory strategies.

## Author Contributions

**Wenjing Zhou:** data curation (lead), formal analysis (lead), investigation (equal), project administration (lead), visualization (equal), writing – original draft (lead). **Yuan Lin:** investigation (lead), resources (lead), supervision (supporting). **Ziwen Liu:** investigation (equal), resources (equal). **Yajing Ma:** data curation (equal), investigation (equal), resources (equal). **Shufeng Hu:** data curation (supporting), investigation (supporting). **Junwu Huang:** data curation (equal), investigation (equal). **Zhou Lu:** supervision (equal). **Lijiang Yu:** methodology (lead), writing – review and editing (equal).

## Funding

The authors have nothing to report.

## Ethics Statement

The animal study research protocol was reviewed and approved by the Animal Care and Welfare Committee of Guangxi University (GXU‐2024‐319). In this study, the bird samples used were all dead at the time of collection, and no living birds were harmed.

## Consent

The authors have nothing to report.

## Conflicts of Interest

The authors declare no conflicts of interest.

## Supporting information


**Data S1:** Sample metadata, including sample IDs, common names, scientific names, Orders, Family, feeding guilds, migratory status, collection dates and collection site.
**Data S2:** Raw abundance table of ASVs across all samples. Abundance table of ASVs with taxonomic classification and prevalence.

**Figure S1:** Rarefaction curves constructed based on the Shannon index.
**Figure S2:** PCoA based on weighted UniFrac (a) and unweighted UniFrac (b) distance among diet groups. CB, carnivore bird; HB, herbivore bird; OB, omnivore bird.

## Data Availability

The sequence data generated during the current study are available in the Sequence Read Archive (SRA) repository under project PRJNA1366654. This data is public: https://www.ncbi.nlm.nih.gov/sra/PRJNA1366654.
