# Supplementary material for: Variation in Bird Gut Microbiota Across Dietary Guilds and Migratory Strategies
Source: Ecol Evol. 2026 Sep 7;16(9):e73879. doi: 10.1002/ece3.73879 (PMC13548137; doi:10.1002/ece3.73879)
Supplement: Supplementary file 1 [file ECE3-16-e73879-s001.docx]

**Fig. S1** Rarefaction curves constructed based on the Shannon index.

**Fig. S2** PCoA based on weighted UniFrac (a) and unweighted UniFrac (b) distance among diet groups. OB, omnivore bird; HB, herbivore bird; CB, carnivore bird.
